# Microbiome Helper: a Custom and Streamlined Workflow for Microbiome Research

**DOI:** 10.1128/mSystems.00127-16

**Published:** 2017-01-03

**Authors:** André M. Comeau, Gavin M. Douglas, Morgan G. I. Langille

**Affiliations:** CGEB-Integrated Microbiome Resource (IMR) and Department of Pharmacology, Dalhousie University, Halifax, Canada; UC Davis Genome Center

**Keywords:** 16S rRNA gene sequencing, Microbiome Helper, bioinformatics, dual-indexing PCR, education, metagenomics, microbiome, standard operating procedure, virtual machine

## Abstract

Sequence-based approaches to study microbiomes, such as 16S rRNA gene sequencing and metagenomics, are uncovering associations between microbial taxa and a myriad of factors. A drawback of these approaches is that the necessary sequencing library preparation and bioinformatic analyses are complicated and continuously changing, which can be a barrier for researchers new to the field. We present three essential components to conducting a microbiome experiment from start to finish: first, a simplified and step-by-step custom gene sequencing protocol that requires limited lab equipment, is cost-effective, and has been thoroughly tested and utilized on various sample types; second, a series of scripts to integrate various commonly used bioinformatic tools that is available as a standalone installation or as a single downloadable virtual image; and third, a set of bioinformatic workflows and tutorials to provide step-by-step guidance and education for those new to the microbiome field. This resource will provide the foundations for those newly entering the microbiome field and will provide much-needed guidance and best practices to ensure that quality microbiome research is undertaken. All protocols, scripts, workflows, tutorials, and virtual images are freely available through the Microbiome Helper website (https://github.com/mlangill/microbiome_helper/wiki).

**IMPORTANCE** As the microbiome field continues to grow, a multitude of researchers are learning how to conduct proper microbiome experiments. We outline here a streamlined and custom approach to processing samples from detailed sequencing library construction to step-by-step bioinformatic standard operating procedures. This allows for rapid and reliable microbiome analysis, allowing researchers to focus more on their experiment design and results. Our sequencing protocols, bioinformatic tutorials, and bundled software are freely available through Microbiome Helper. As the microbiome research field continues to evolve, Microbiome Helper will be updated with new protocols, scripts, and training materials.

## Abstract (precis)

As the microbiome field continues to grow, a multitude of researchers are learning how to conduct proper microbiome experiments. We outline here a streamlined and custom approach to processing samples from detailed sequencing library construction to step-by-step bioinformatic standard operating procedures. This allows for rapid and reliable microbiome analysis, allowing researchers to focus more on their experiment design and results. Our sequencing protocols, bioinformatic tutorials, and bundled software are freely available through Microbiome Helper. As the microbiome research field continues to evolve, Microbiome Helper will be updated with new protocols, scripts, and training materials.

## INTRODUCTION

Research has associated the human microbiome with lifestyle changes (1), severity of disease (2–4), treatment outcomes (5, 6), and the built environment (7). Meanwhile, the microbiomes from oceans (8) to atmospheres (9) have provided key insights into the influence and response of particular microbes to various complex environments.

Methods for profiling microbiome samples have changed from presequencing techniques, such as denaturing gradient gel electrophoresis and terminal restriction fragment length polymorphisms, to newer sequenced-based approaches, including amplicon rRNA gene sequencing, metagenomics, and metatranscriptomics. These sequencing approaches remain challenging and complex for both library preparation and bioinformatic analysis, especially for those researchers just entering the microbiome research field. Currently, Illumina sequencing is the most prevalent next-generation sequencing (NGS) technology for microbiome sequencing, and several studies have outlined different methods and strategies for processing samples (10–12). Multiplexing (“barcoding”) of samples was initially conducted using a single-indexing strategy (10) and then later replaced by a dual-indexing strategy (11) that leverages barcodes on both the forward and reverse paired ends (PE). At one point, Illumina sequencing had difficulties with low-sequence-complexity libraries like those resulting from 16S rRNA gene profiling, and a large proportion of phiX control DNA was spiked in to improve sequencing quality. An alternative approach that used heterogeneity spacers within the barcode provided an improvement to low sequence diversity (12) but required custom barcodes and more-complicated demultiplexing during bioinformatic analysis. Illumina has since improved its sequencing image base-calling to require less phiX, even with very low sequence diversity libraries, opening the door for a simplified dual-indexing strategy.

On the bioinformatics front, there have been tremendous efforts to develop systems to process and analyze microbiome data, including QIIME (13) and mothur (14), primarily for 16S rRNA data, while metagenomic data analysis is still often pieced together using various individual bioinformatic methods (15). Microbiome analysis is ever-changing, and currently, users require training on the myriad of options within and between various bioinformatic tools. Some bioinformatic developers have made efforts to publish tutorials (13) and standard operating procedures (SOPs) (11) to help guide researchers into best practices, but these are often limited to a single tool or platform and usually do not include complete end-to-end guidelines.

Here, we provide an open, user-friendly, and streamlined SOP for both microbiome sequencing and bioinformatic analysis. Our approach has been tested, debugged, and refined on over 17,000 samples processed through Dalhousie University’s Integrated Microbiome Resource (IMR; http://cgeb-imr.ca) and is continually updated with the most recent modifications. Bioinformatic resources including SOPs, custom scripts to allow easier data interoperability and parallelization, comprehensive tutorials, and a VirtualBox image are accessible through Microbiome Helper.

## RESULTS AND DISCUSSION

### IMR run performance.

**FIG 1:**
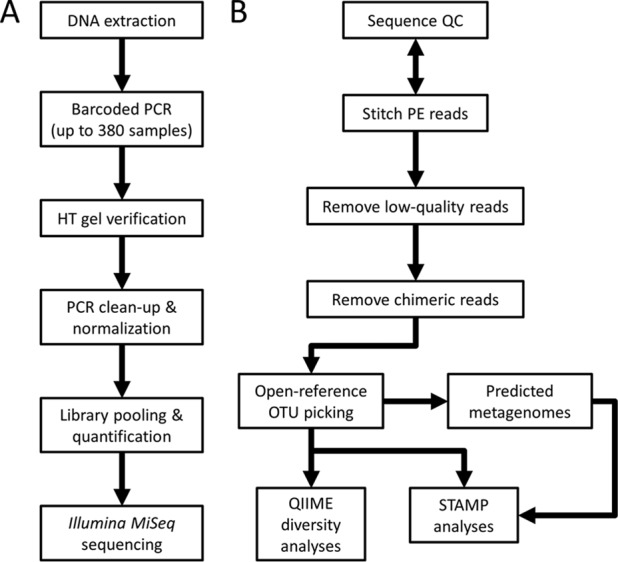
Workflow diagram of on-bench and bioinformatic custom pipelines. Only major steps for both the sequencing protocol (A) and bioinformatics protocol (B) are illustrated. HT, high-throughput; PE, paired-end; QC, quality control.

Amplicon samples in our presented protocol are run on the Illumina MiSeq using 2 × 300 bp PE v3 chemistry which allows for overlap and stitching together of paired amplicon reads into one full-length read of higher quality (see below for further quality discussion). As of December 2016 at the IMR, we have processed over 17,000 samples using this workflow, and we present an overview of the results of our first 25 runs in Table 1. The step-by-step detailed lab protocol is included as supplemental methods in Text S1 in the supplemental material, and a general overview of the workflow is presented in Fig. 1. As presented further below for the bioinformatics approaches, the open lab protocol will be continuously revised/refined as new molecular approaches are introduced by the community, or novel products arrive in the marketplace, and we have had the opportunity to validate them.

10.1128/mSystems.00127-16.1 TEXT S1  Detailed microbiome amplicon sequencing protocol from raw DNA to Illumina MiSeq sequencing. Download TEXT S1, PDF file, 1.8 MB. Copyright © 2017 Comeau et al. 2017 Comeau et al. This content is distributed under the terms of the Creative Commons Attribution 4.0 International license.

**TABLE 1:** Run metrics for the first 25 amplicon runs (9,145 total samples) at the IMR and comparison to Illumina’s MiSeq maximum output specifications (for phiX)

Run^a^	No. of samples	Cluster density (1,000/mm^2^)	Pass filter %	% phiX	% >Q30	% error	Bases (Gb)	Raw reads (million)	Pass filter (million)
IMR1	95	1,019	89	4.9	70	2.8	14.1	25.9	23.0
IMR2	384	938	91	3.9	78	2.7	13.3	23.9	21.7
IMR3	382	1,026	89	5.5	77	2.6	14.1	25.9	23.0
IMR4	376	865	90	6.7	76	2.7	12.1	21.8	19.6
IMR7	372	1,030	88	5.5	67	2.7	14.1	26.1	22.9
IMR15	376	585	93	3.9	63	3.7	8.8	15.4	14.3
IMR16	387	840	91	4.4	75	2.9	11.9	21.4	19.5
IMR17	380	949	90	4.3	64	3.4	13.3	24.1	21.6
IMR19	380	730	91	5.7	74	2.9	10.2	18.3	16.7
IMR20	377	893	90	4.3	65	3.8	12.4	22.4	20.1
IMR21	380	914	91	3.6	70	3.9	12.8	22.9	20.9
IMR22	380	926	94	4.3	69	3.0	13.6	23.5	22.2
IMR23	380	837	91	5.6	70	2.8	11.8	21.1	19.3
IMR24	380	732	92	3.7	64	2.9	10.7	18.9	17.5
IMR25	380	989	89	5.2	70	2.4	13.7	25.1	22.3
IMR26	379	938	95	6.4	69	2.4	14.0	24.0	22.8
IMR27	376	1,013	86	5.6	57	2.4	13.1	24.8	21.3
IMR28	363	845	92	7.5	81	2.1	11.9	21.1	19.4
IMR29	360	893	90	14.7^b^	81	2.6	12.1	22.0	19.8
IMR30	380	960	88	5.6	74	2.2	13.0	24.1	21.2
IMR31	377	845	91	5.5	76	2.2	11.9	21.5	19.4
IMR32	373	805	91	4.8	80	2.1	11.2	20.2	18.3
IMR34	380	905	93	4.9	69	2.3	13.2	23.3	21.6
IMR35	380	1,112	90	3.9	72	2.3	15.5	28.0	25.3
IMR36	368	982	88	2.4	62	2.8	13.0	23.9	21.1
									
Mean	NA^c^	903	91	4.9	71	2.7	12.6	22.8	20.6
Illumina	NA	1,200–1,400	NA	NA	>70	NA	13.2–15.0	NA	22–25

a Numbering is not consecutive as other (metagenomics) runs were completed in between amplicon runs.

b Extra phiX was added to this run for diagnostic reasons and does not factor into the below mean.

c NA, not applicable.

We consistently aim for the Illumina-recommended 20 pM library loading with an accompanying 5% phiX, which is now the minimal amount to maintain high-quality base-calling for low-diversity (amplicon) libraries (16). Average cluster densities of ~900,000/mm^2^ produced final outputs of ~21 million pass-filter reads (~13-Gb raw data) at a Q30 (bases with a quality score of at least 30) of 71%. These values equate to ~55,000 raw reads per sample for a typical complete run of 380 samples. Note that achieved cluster densities and final outputs are slightly below Illumina’s “best-case scenario” specifications, but this is to be expected as the latter is for a complex phiX genomic library, whereas the former amplicon libraries typically pose a challenge for NGS systems and can easily overcluster (saturate), leading to reduced Q30 and pass-filter read numbers.

**FIG 2:**
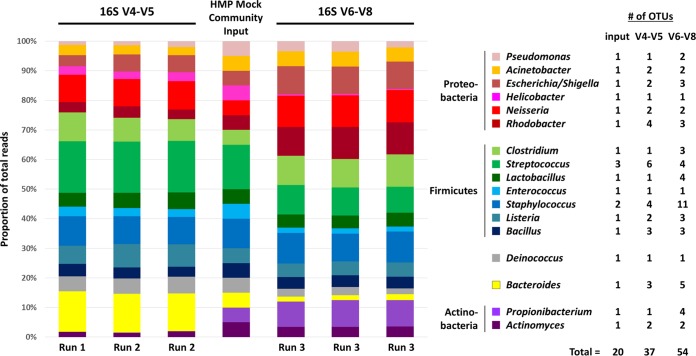
Example of amplicon performance in our presented workflow. The bacterial HMP Mock Community “B” sample (BEI Resources) was processed using 16S V4-V5 (515F+926R [28]) and V6-V8 (B969F+BA1406R [27]) primer sets through our on-bench and bioinformatics workflow. Three replicates for each region were sequenced on three independent MiSeq runs and then normalized to 15,000 reads each during analysis. The mock sample contains equal amounts of rRNA gene copies of 20 species, one for each of the genera listed in the legend, except two species of *Staphylococcus* and three species of *Streptococcus*. Note that the individual variable regions have difficulty separating *Escherichia* from *Shigella* and that they are listed with interchangeable identities here. The output numbers (always 37 or 54) and identities of the OTUs were consistent between each replicate of a given variable region.

For diagnostic purposes, and to show an example of sample performance, the commonly used bacterial Mock Community “B” developed for the HMP project (BEI Resources) was processed using our protocol, employing two separate sets of primers for the V4-V5 and V6-to-V8 regions of the 16S rRNA gene (Fig. 2), in triplicate on three independent sequencing runs. The sample contains equal amounts of rRNA gene copies of 20 bacterial species, one for each of the genera listed in the figure, except for *Staphylococcus* and *Streptococcus*, which have 2 and 3 species each, respectively. We include here the results from two different 16S variable regions as a reminder to the uninitiated that not all variable regions (within 16S, 18S, or internal transcribed spacer [ITS]) are created equal—different results will be obtained from the same starting material depending on your choice of target and specific primer pairs used. Due to these differences, it is also difficult to compare independent studies that have used different variable regions and thus this will also guide researchers to their choice of region/primers if they wish to compare their results to previous work in their field (or wish to study specific taxa that have severe biases with certain primer pairs). Various *in silico* and *in vitro* examinations of region differences (see references 17, 18, and 19 for examples) have highlighted that two prime reasons exist for these differences: (i) primer amplification efficiency in PCR, due to binding strengths directly at the site of annealing or downstream secondary structure effects on polymerase extension, can cause misrepresentations of different sequences (species/strains) in the final fragment pool (i.e., some amplify more easily or poorly than average) and (ii) not all taxonomic groups have the same degree of resolution in each of the regions, meaning that, even if amplification is successful, downstream clustering into operational taxonomic units (OTUs) (at a set identity level for all, such as 97%) and identification will be hampered in some groups. In our present example, the V4-V5 region overrepresents *Firmicutes* and *Bacteroides* while severely underestimating *Actinobacteria*; *Propionibacterium* nearly disappears with 7 to 10 reads versus 750 expected. Conversely, the V6-to-V8 region shows more accurate proportions of *Actinobacteria* and *Firmicutes* but overestimates *Proteobacteria* while at the same time having difficulty with *Bacteroides* and *Helicobacter* (the latter being at ~10% of the expected value). Both regions overestimate species richness: ~2- to 3-fold-more 97% identity OTUs were found for the 20 species present in the mock community. However, these details are not unexpected, and our intent is not to present an in-depth comparative analysis of variable regions or richness estimates, as these topics have been covered in multiple previous studies, as mentioned above. More importantly, the results show that reproducibility is high with very consistent replicates within each variable region. Sequence proportions show very low coefficients of variation (10.9% for V4-V5 and 6.4% for V6 to V8), and the exact same OTUs are found each time, indicating a robust library preparation and sequencing protocol that we can recommend for general use. Users can select the variable region of their choice for use in our protocol, and we will be updating our default choices within Microbiome Helper as the state of the art in region choice and primer sequences evolves within the community.

### Pipeline computational and sequence quality performance.

As an example of our bioinformatics pipeline, we reanalyzed 16S rRNA gene V6-to-V8 sequencing data extracted from 116 mouse fecal samples (part of IMR7 in Table 1). This data set was generated to compare the microbiomes of chemerin-knockout strains compared to wild-type strains and serves as our example tutorial data on the Microbiome Helper wiki. For each major step, we computed the time required to run the process on 1 central processing unit (CPU) (estimated by the sum of the “user” and “sys” times outputted by the “time” command), the number of PE reads remaining, and the number of OTUs (Table 2). The entire pipeline was threaded over 30 CPUs where possible and ran in 61 h of CPU time, which translated to 5.5 h in real time. Open-reference OTU picking was the lengthiest step (38 h of CPU time), followed by chimera removal (17 h of CPU time).

**TABLE 2:** Run times and data metrics of a 16S Microbiome Helper run

Process^a^	CPU time^b^ (h:min)	No. of reads (10^6^)	% reads remaining	No. of OTUs
FastQC report	0:13	7.5	100.0	NA^c^
Stitch reads	3:22	7.4	98.6	NA
Filter reads	1:31	4.7	62.2	NA
Remove chimeras	17:08	4.2	56.1	NA
Pick open-reference OTUs	38:01	4.2	56.1	139,253
Remove low-confidence OTUs	0:01	3.8	51.3	4,504
Sample rarefaction	0:02	1.4	19.2	4,504
Beta-diversity plot	0:01	1.4	19.2	4,504
Alpha-diversity plot	0:25	1.4	19.2	4,504

a The commands used are described here at https://github.com/mlangill/microbiome_helper/wiki/16S-standard-operating-procedure.

b These are the run times for one 2.3-GHz CPU (the time was 5.5 h in real time on 30 of the same CPUs).

c NA, not applicable.

The vast majority (98.6%) of read pairs were successfully stitched, and this was consistent across all samples (range of 98.0 to 98.9%). After read filtering based on quality and length, as well as chimera removal, 56.1% of reads were still retained. We used default filtering options for both steps (see Materials and Methods). The percentage of reads discarded by both the quality/length filtering and chimera removal steps was variable across samples—ranging from 33.0 to 45.6% and from 4.3 to 19.3%, respectively. Our preference is to be stringent on read quality to avoid generation of spurious OTUs downstream; however, quality and length filtering can be changed easily by the user depending on amplicon length or tolerance of lower-quality reads.

**FIG 3:**
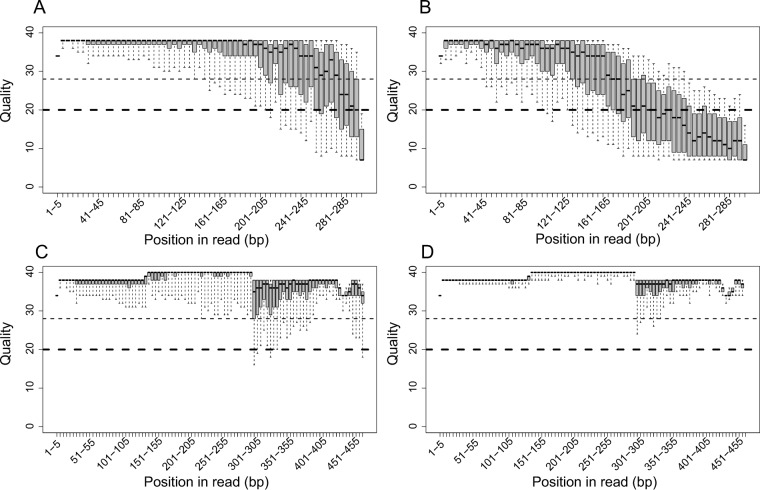
Box plots of quality scores over positions in sequenced reads. Quality distributions are shown for forward reads (A), reverse reads (B), raw stitched reads (C), and filtered stitched reads (D) from a single MiSeq run. These plots are adapted from the FastQC output: the thick and thin dashed lines indicate quality scores of 20 and 28, respectively (to match the default FastQC output).

Fully overlapping read pairs have previously been recommended for 16S rRNA analyses, since the increase in sequencing errors in nonoverlapping regions can result in spurious OTU calls (11). Related to this suggestion, there has also been some concern about the base-quality performance of Illumina’s v3 kit chemistry toward the ends of the 300-nucleotide (nt) reads (20). These are valid concerns, and we have tweaked our quality-filtering steps to help minimize these problems. The distribution of quality scores in forward and reverse reads is shown in Fig. 3A and B to help visualize this issue. For both forward and reverse reads, there is a decrease in quality near the 3′ ends. After stitching these 300-nt reads together (Fig. 3C), there is a clear increase in quality in the overlapping region (from ~150 to 300 bp). Notably, there is a large degree of variation in quality, especially toward the end of the reads. After quality filtering, there is much less variation (Fig. 3D). Despite this improvement in quality score distributions, spurious OTU calls still occur, as shown by the massive number of raw OTUs originally called (139,253). After eliminating OTUs that are called by <0.1% of reads (our “remove low-confidence OTUs” step), which is the maximal expected bleed-through between MiSeq runs according to Illumina (21), we retained 4,504 OTUs. This 97% removal of (most probably) spurious OTUs demonstrates, along with the mock community results in Fig. 2, that reasonable OTU estimates can be achieved by using proper quality control.

### Application of workflows and tutorials.

Microbiome Helper provides suggested workflows or SOPs for 16S, 18S, ITS2, and metagenomic analysis, from raw data through visualization and statistics. These SOPs provide step-by-step explanations of every Unix command along with details about what the command is doing and what options the users may want to change when using their own data set. As in many research fields, there is not often a clear single “right way” to process microbiome data, and so when possible, we attempt to document alternative approaches. For example, in addition to the open-reference OTU clustering process, we provide several emerging alternatives that attempt to model Illumina sequencing error to allow for greater resolution beyond 97% OTUs (22–24). In addition to SOPs, tutorials with premade data sets and example outputs are provided for 16S analysis, metagenomic taxonomy, metagenomic function, PICRUSt inference, and visualization and statistical analysis with STAMP. Methods that attempt to predict phenotypic information from OTU tables such as BugBase (25) and FAPROTAX (26) are also included in the Microbiome Helper wiki and virtual image. These tutorials are not just lists of “copy-and-paste” commands but rather aim to educate the researcher by explaining what is actually being conducted and also contain questions at various steps (with answers provided on separate pages). All workflows and tutorials are easily run within the Microbiome Helper virtual image, which combines all necessary bioinformatic packages and avoids complicated and timely installation. The bioinformatic SOPs have been rigorously tested both in-house and with several collaborators on thousands of 16S and 18S rRNA samples and hundreds of metagenomic samples. The tutorials and virtual image have been deployed at four different workshops and have been used by hundreds of trainees at various experience levels in the microbiome field. All scripts, workflows, and tutorials are freely available and continually updated in response to changing methods and approaches. We encourage other educators to incorporate these tutorials into their training environments and would gratefully include tutorials from others into Microbiome Helper.

### Conclusion.

As the microbiome field continues to rapidly expand, there is a great demand for clear, concise, and well-tested protocols for both sequencing and bioinformatic analysis. It is unlikely that the entire field will agree to the exact same workflows, due to differences in scientific interests and difference of opinions on optimal methods. Here, we have presented a set of protocols, workflows, and tutorials that has been shown to produce reliable and consistent results across a variety of samples and has been already successfully deployed as a training resource. Microbiome Helper is freely and openly available and will continue to evolve as the field grows.

## MATERIALS AND METHODS

### Amplicon library preparation and sequencing.

The following subsections summarize the generation of PE sequencing reads of 16S or 18S rRNA gene PCR amplicons with multiple barcodes (indices) on the Illumina MiSeq machine of a length of approximately 400 to 500 bp. It assumes an input of up to 384 slots (380 samples plus 4 PCR-negative controls) conducted in four 96-well plates and can be done manually or using liquid-handling robotics. These bench protocols are a synthesis of multiple sources in the scientific literature as to the current “best practices” but draw heavily upon the work of Comeau et al. (27) for initial primer design and PCR setup. Here, we present the examples of 16S V6-to-V8 (bacteria and archaea) and 18S V4 (eukarya) amplicons, but the protocols can be easily modified for use with any correctly sized amplicon(s) of your choice—such as other rRNA gene variable regions or any functional genes of interest (such as *psbA*, *cox1*, etc.). At the IMR, we have successfully tested and deployed additional amplicons for 16S V4-V5 (28), fungal ITS1 and ITS2 (28, 29), nitrogen cycle *nifH* (J. Laroche, unpublished data), and BarSeq mutant analysis (30).

### (i) Custom Illumina primers.

**FIG 4:**
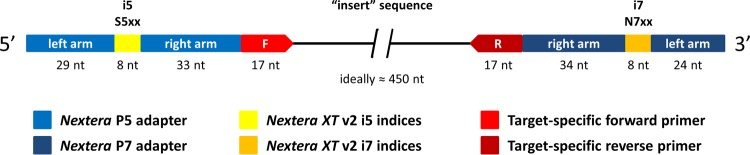
Diagram of fusion primers and dual-indexing approach for library construction. Illumina Nextera left (P5) and right (P7) adaptors contain the i5 (S5xx series) and i7 (N7xx series) indices in their respective middle sections, respectively. All nucleotide sizes within fusion primers are the same for different target amplicons, with the exception of the target-specific primer regions (F+R) which here demonstrate the sizes of the 16S rRNA gene V6-V8 primers (B969F+BA1406R [27]) employed in the presented protocol.

A dual-indexing, one-step PCR is done using complete “fusion primers” that include Illumina Nextera adaptors plus indices plus specific regions targeting either the 16S or 18S rRNA genes (Fig. 4). We utilize all of the Nextera v2 set A to D indices: 16 forward × 24 reverse indices means that, with only 40 different fusion primers, all 384 combinations can be achieved (see Text S1 in the supplemental material for the layout of the indices). A spreadsheet template is provided in Table S1 with the sequences of the fusion constructs currently employed at the IMR and the capacity to plug in any specific primers, targeting genes/regions of your choice, to create your own primers compatible with this protocol and Illumina’s sequencing technology. The one-step approach is in contrast to a two-step protocol whereby a first PCR is conducted using specific primers, followed by a second “indexing” PCR using a separate indexing kit/set of primers that fuses adaptors plus (single or dual) barcodes to the sample amplicons. Although still an option for those who wish to decouple specific primers from barcodes, we prefer the one-step approach as there are multiple advantages: (i) simpler logistics, having one primer combination per sample well from beginning to end, mitigating chances for error; (ii) reduced chances of chimeric PCR product formation and compounded amplification biases by avoiding second-round PCR; and (iii) reduced costs by avoiding extra verification plus cleanup steps and requiring less of the PCR reagents per sample.

10.1128/mSystems.00127-16.2 TABLE S1  Custom fusion primer templates for multiplexing various amplicon regions up to 384 samples per sequencing run. Download TABLE S1, XLSX file, 0.04 MB. Copyright © 2017 Comeau et al. 2017 Comeau et al. This content is distributed under the terms of the Creative Commons Attribution 4.0 International license.

### (ii) PCR, cleanup, and normalization.

16S or 18S rRNA gene fragments are PCR amplified from the DNA in duplicate using separate template dilutions (generally 1:1 and 1:10) using a high-fidelity polymerase (critical to reduce spurious diversity in amplicons). For the generation of 18S amplicons from microbiome samples containing substantial nontarget host DNA (e.g., human, mouse, etc.), we employ modified Earth Microbiome Project recommendations for using a mammalian blocking primer—note that their protocol employs a V9 blocking primer since their amplicons are generated for the V9 region but that our protocol uses the longer V4 region for amplicons, and hence, we use a V4 blocking primer to match (see reference 31 and also Text S1 in the supplemental material). Four negative PCR controls are included on every run (1 per 96-well plate), and occasionally when significant changes are made to the protocol, a positive control is also included. As shown in the results above, we originally used the HMP Mock Community “B” (evenly distributed composition, catalog no. HM-782D) supplied freely to noncommercial researchers from BEI Resources as a positive control, but the product has since been discontinued. However, there are now commercial alternatives available, such as the ZymoBIOMICS standards from Zymo Research (Irvine, CA). The duplicate PCRs are combined in one plate and then verified visually by running a high-throughput Invitrogen 96-well E-gel. Any samples with failed PCRs (or spurious bands) are reamplified by optimizing PCR conditions to produce correct bands in order to complete the sample plate(s) before continuing. Amplicons are then cleaned up and normalized in one step using the high-throughput Invitrogen SequalPrep 96-well plate kit. The (up to) 380 samples plus 4 negative controls are finally pooled to make one library which is then quantified using the Invitrogen Qubit double-stranded DNA high-sensitivity (dsDNA HS) fluorescence-based method before sequencing.

### (iii) On-machine custom run setup.

As neither the Illumina MiSeq Control Software (MCS) nor Experiment Manager (iEM) software typically accepts/expects libraries over 96 index combinations, some minor manual “hacking” of the sample sheets is required for our custom application to be loaded correctly. The supplemental materials and methods in Text S1 contain detailed instructions for preparing these sample sheets. It is currently only a lack of support from Illumina on the run-prep software side that leads to this work-around; the v3 kit chemistry fully supports physically sequencing these libraries, and Illumina’s cloud-based BaseSpace application also properly handles postrun delivery and analysis of these samples.

### Bioinformatics pipeline.

**FIG 5:**
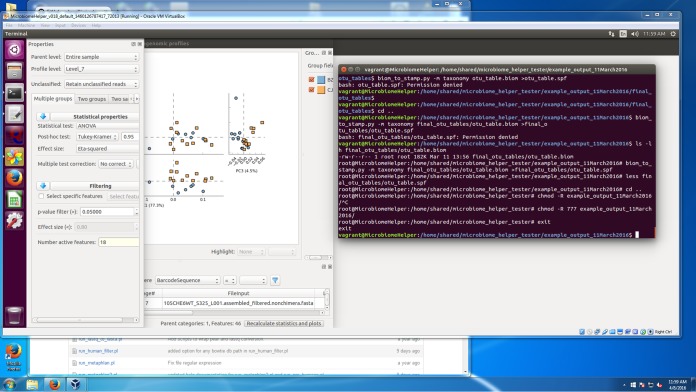
Screenshot of the Microbiome Helper virtual image. The screenshot illustrates the Ubuntu-based Microbiome Helper virtual image running within a Windows 7 operating system with both a terminal window for command-line access and the graphically based STAMP visualization and statistics tool.

We have produced a straightforward and detailed pipeline called Microbiome Helper that utilizes many publicly available tools to perform the major steps of 16S rRNA analysis (see the workflow in Fig. 1). Where necessary, we have written wrapper scripts to allow multiple samples to be run simultaneously and to seamlessly integrate multiple tools by correcting for file format differences. These scripts are written typically in either Perl or Python and are available at the Microbiome Helper website. In addition, these tools have been bundled in an Ubuntu 16.04 VirtualBox image which will allow the steps described below to be performed on a 64-bit personal computer (Windows/Mac OS X/Linux) with little or no configuration (see screenshot in Fig. 5) and only modest specifications (e.g., 8 GB random-access memory [RAM], 2 cores, etc.).

### (i) Preprocessing.

FastQC (v0.11.5 [32]) is used to evaluate the quality of raw PE reads, which is useful for identifying problematic sequencing runs and/or samples. Next, stitching of unambiguous read pairs together is done using PEAR (v0.9.10 [33]). To confirm that read stitching has been performed correctly, we again run FastQC on the stitched reads. Examining the quality and size distributions of the stitched reads enables sensible cutoffs to be chosen for the read-filtering step. Based on these cutoffs, FASTX-Toolkit (v0.0.14 [34]) is used to filter out reads that have more than a specified proportion of low-quality sites, with the default in our SOP being a quality score of 30 over at least 90% of the bases. We then filter out reads shorter than a specified length (<400 bp by default) and reads that do not exactly match the known primer sequences at the 5′ and 3′ ends using BBMap (v35.85 [35]). These quality thresholds can be set by the user since optimal settings will differ for each data set. Following read filtering, we screen out potentially chimeric reads using VSEARCH (v1.11.1 [36]), which implements the UCHIME algorithm (37). By default, we use the options mindiv=1.5 and minh=0.2 for chimera checking. Where necessary, scripts have been written to execute these steps over multiple samples at once to leverage parallel computing and to avoid repetitive commands.

### (ii) OTU picking, spurious data removal, and taxonomic assignment.

We use QIIME wrapper scripts (v1.91 [13]) to classify reads into different operational taxonomic units (OTUs; at 97% identity for 16S and 98% for 18S), which is called OTU picking. Specifically, we run open-reference OTU picking, which means that reads are first clustered against reference sequences and then any remaining reads are clustered against themselves (*de novo*) (38). To avoid memory limitations as a result of closed-source 32-bit clustering methods, we opted for inclusion of open-source methods SortMeRNA (v2.0-dev time stamped 29/11/2014 [39]) and SUMACLUST (v1.0.00 [40]) for the reference-based and *de novo* clustering steps, respectively. Running both of these steps is important, since using reference-based methods alone can lead to biases in OTU picking (41). To remove spurious OTUs that are a result of unfiltered chimeras or “bleed-through” between sequencing runs, a dynamic cutoff (as opposed to removing just singletons) is employed to filter out OTUs having <0.1% of the total number of sequences. The OTU table is then normalized per sample by subsampling (or rarefying) to a minimal number of reads, but we also provide specific details on how DESeq2 (42) can alternatively be used to statistically normalize the table without the loss of data (43).

### (iii) Additional analyses and visualization.

After the final OTU table is created, Microbiome Helper provides numerous options for analysis. This includes details on how to use QIIME to calculate alpha- and beta-diversities, to run principal-coordinate analysis on UniFrac distances (44), and to test for statistical differences between groups (13). Scripts are provided to convert BIOM-formatted OTU tables to other formats such as STAMP (v2.1.3 [45]), which is used to identify particular taxa that significantly differ in abundance between groups, as well as for several visualizations. Detailed steps on the use of PICRUSt (v1.1.0 [46]) are provided to infer the functional content of samples in terms of KEGG orthologs and pathways and to associate taxonomic changes with functional differences.

Scripts are also provided for metagenomic bioinformatic analysis, including wrapper scripts for MetaPhlAn2 (47), HUMAnN (48), and Kraken (49), and integration of these tools with STAMP.

### (iv) Workflows and tutorials.

Standard operating procedures or workflows are provided for both 16S rRNA and metagenomic bioinformatic analysis within the Microbiome Helper wiki. These provide step-by-step guides for each command to be run with a brief explanation of its purpose and what options may need to be changed depending on the specific data set. In addition, several tutorials from previous workshops, including the Canadian Bioinformatics Workshop in Analysis of Metagenomic Data (hosted by bioinformatics.ca), Strategies and Techniques for Analyzing Microbial Population Structures (hosted by the Marine Biological Laboratory), Metagenomics Workshop (hosted by the Great Lakes Bioinformatics conference), and Metagenomics Bioinformatics (hosted by the European Bioinformatics Institute), are available on the Microbiome Helper wiki. These tutorials include practice data sets, detailed descriptions of each command, explanations of output, examples of visualizations, and questions to prompt interactive learning. These tutorials can all be completed using the Microbiome Helper VirtualBox image.

### Ethics approval and consent to participate.

All protocols were conducted in accordance with the Canadian Council on Animal Care guidelines and approved by the Dalhousie University Committee on Laboratory Animals.

### Availability of data and material.

The data sets and scripts supporting the conclusions of this article are available in the Microbiome Helper repository (https://github.com/mlangill/microbiome_helper/wiki).
