# Supplementary material for: Microbiome Helper: a Custom and Streamlined Workflow for Microbiome Research
Source: mSystems. 2017 Jan 3;2(1):e00127-16. doi: 10.1128/mSystems.00127-16 (PMC5209531; doi:10.1128/mSystems.00127-16)

Supplementary Methods for:

Microbiome Helper: A custom and streamlined workflow for microbiome research
A. M. Comeau, G. M. Douglas and M. G. I. Langille

Microbiome Amplicon Sequencing Workflow

The following detailed protocol is for the generation of paired-end sequencing reads of 16S or 18S PCR amplicons with multiple barcodes (i.e.: “indices”) on the *Illumina MiSeq* machine of length ≈ 450 bp (300+300 bp with ~150 bp overlap) using v3 chemistry. It assumes an input of 380 individual samples (+4 controls = 384) in four 96-well plates (384-well plates are discouraged). Catalog numbers are given when items from specific vendors (vs. generic choices) are required.

Required Equipment

- Single-channel pipettes (2-1000 μL)
- Multi-channel (8) pipette(s) (MCP; 2-100 μL)
- Required equipment for the DNA extraction kit(s) of your choice
- PCR machine with 96-well block
- *Invitrogen Mother E-Base* (#EB-M03) for *E-Gels 96*
- Documentation system for DNA gels (SYBR Safe filter)
- Microvolume DNA fluorescence reader (such as *Invitrogen Qubit*)
- *Illumina Experiment Manager (iEM)* software
- *Illumina MiSeq* sequencer with *RTA v1.17.28* / *MCS v2.2* or later

Optional/Case-Dependant Equipment

- Bead-mill / TissueLyser / Homogenizer (if using bead-based DNA extractions)
- Microvolume DNA spectrophotometer (such as *NanoDrop*; for quantifying extracts)
- Centrifuge with rotor for 96-well plates (useful for spinning down condensation)
- Standard gel electrophoresis system (for analyzing recalcitrant samples)

Required Reagents & Consumables

- Pipette filter tips (p2 to p1000)
- Reagent reservoirs for MCP
- 1.5 mL Eppendorf tubes
- DNA extraction kit(s)
- 96-well thin-wall 0.2 mL PCR plates (such as *Bio-Rad* #HSP9601)
- PCR plate films (such as *Bio-Rad* #MSB1001)

- *Thermo Phusion High-Fidelity DNA Polymerase* (#F-530L) or similar
- dNTP mix (at 40 mM = 10 mM of each base)
- *Illumina* fusion primers (see below and Excel template)
- PCR-grade water
- *Invitrogen E-Gels 96 2% with SYBR Safe* (#G7208-02)
- *Invitrogen E-Gel Low Range Ladder* (#12373-031; diluted 1:1 with PCR-grade water)
- *Invitrogen SequalPrep Normalization Kit* (#A10510-01)
- *Invitrogen Qubit dsDNA HS* reagent and assay tubes (clear 0.2 mL)
- 2 N NaOH
- 200 mM Tris-HCl, pH 7
- *Illumina PhiX Control Kit v3* (#FC-110-3001)
- *Illumina MiSeq Reagent Kit v3 (600 cycle)* (#MS-102-3003)

Optional/Case-Dependant Reagents & Consumables

- Ethanol (usually a requirement for extraction/purification kits)
- Mammalian blocking primer (see **viii** below; if substantial host contamination in 18S)
- Thin-wall 0.2 mL PCR tubes or strips with caps (for re-amplifying recalcitrant samples)
- Agarose, loading buffer/stain and 100 bp ladder (for analyzing recalcitrant samples)
- PCR product purification kit (if need to concentrate final library)

Advance Preparation – Order and Aliquot Primers (1 h)

i Use our Excel template to copy existing 16S/18S/ITS primers or to design your own custom gene primers with the proper Illumina indices and Nextera adaptor orientations. We order IDT "Ultramers" (www.idtdna.com) for such long primers (~80-90 nt) as their coupling efficiency is one of the highest available (critical for obtaining high proportions of full-length oligos in the mix you obtain). Order the fusion primers at 4 nmole scale in deep-well plates; one set per 96-well plate, arranged as follows, leaving blank rows in between sets:

S502	S503	S505	S506	S507	S508	S510	S511	←	Forward Set 1		
S513	S515	S516	S517	S518	S520	S521	S522	←	Forward Set 2		
N701	N702	N703	N704	N705	N706	N707	N710	N711	N712	N714	N715
								↖	Reverse Set 1		
N716	N718	N719	N720	N721	N722	N723	N724	N726	N727	N728	N729
								↖	Reverse Set 2		

ii Once arrived, add 400 µL of PCR-grade water to each well containing the primers in order to reconstitute them at a concentration of 10 µM (1/10th the typical 100 µM working stock concentration for primers). We have found that these usually need a significant incubation time for the lyophilized pellets to re-suspend – we typically leave them overnight at 4°C.

iii Prepare the 1 µM working stock **Forward Set 1 Primer Plate** by pipetting 63 µL of PCR-grade water into each well of the 96-well PCR plate from a sterile reservoir. Rotate the deep-well primer plate 90° clockwise and align it so that the 8 occupied wells (= 8 different indices) of row 1 line up with the 8 rows of the new plate. Working by column and keeping the same set of tips, transfer 7 µL of reconstituted primer into each well of each column, mixing well by pipetting. Once complete, each column of the resulting plate will have enough primer for one complete 96-well plate PCR (leaving some extra for pipetting error; 12 columns × 5 µL = 60 µL required). Seal the plate with PCR film and store at -20°C.

iv Prepare the 1 µM working stock **Forward Set 2 Primer Plate** by repeating step **iii**, but using row 3 of the reconstituted deep-well primer plate.

v Prepare the 1 µM working stock **Reverse Set 1 Primer Plate** by pipetting 45 µL of PCR-grade water into each well of the 96-well PCR plate from a sterile reservoir. Align the deep-well primer plate horizontally (normal orientation) so that the 12 occupied wells (= 12 different indices) of row 5 line up with the 12 columns of the new plate. Working by row and keeping the same set of tips, transfer 5 µL of reconstituted primer into each well of each row, mixing well by pipetting. Once complete, each row of the resulting plate will have enough primer for one complete 96-well plate PCR (leaving some extra; 8 rows × 5 µL = 40 µL required). Seal the plate with PCR film and store at -20°C.

vi Prepare the 1 µM working stock **Reverse Set 2 Primer Plate** by repeating step **v**, but using row 7 of the reconstituted deep-well primer plate.

vii Once all aliquoting is complete, seal the deep-well plate with PCR film and archive at -20°C until new aliquots are required (minimized freeze-thaw cycles).

viii *Optional: For the generation of 18S V4 amplicons from microbiome samples containing substantial non-target host DNA (ex: human, mouse, etc.), order a custom PNA mammalian blocking primer (elongation arrest in the V4 region courtesy of Laura Parfrey and Matt Lemay [UBC]) with the sequence: 5'-TCTTAATCATGGCCTCAGTT-3'. Once arrived, prepare an archival stock of 100 µM and a working stock of 10 µM using PCR-grade water.*

Step 1 – DNA Extraction (1 day)

1.1 Extract DNAs from your samples using the method/kit appropriate to the specific samples (ex: generally stool [with bead-beating kits], but also urine, etc.). For example, we have had good success with the *MO-BIO PowerFecal Kit* for mouse pellets

and human stool. *Note: Do not overload the kit columns with excessive amounts of sample material as this reduces extraction efficiencies and can allow contaminants/inhibitors to co-purify with the DNAs.*

1.2 Quantify and quality-check your final DNAs via *NanoDrop* or *Qubit/PicoGreen* to verify success. The $A_{280/260}$ ratios should be 1.8 or better and our experience with community DNAs has shown that a concentration of at least 1 ng/µL is required to get consistent PCR results. Avoid costly secondary extractions/clean-ups for inhibitors (unless obvious) until PCRs below have truly shown inhibition – many sub-optimal DNAs will still perform adequately in PCR. *Optional: A gel can be run to verify integrity (generally unnecessary for PCR-only studies).*

1.3 Aliquot 5 µL of each of the 380 DNA samples into 4 × 96-well PCR plates (**DNA Plates 1-4**) in the order desired (we prefer by column), allowing for one PCR negative control well (position H12) on each plate; the layout of the primers (for ex: showing F1+R2 sets are used for Plate 2) are also indicated on this figure:

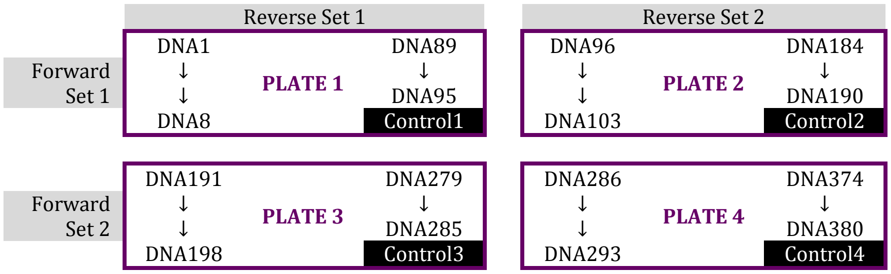

Step 2 – 16S or 18S PCR (1 day)

2.1 Prepare the following PCR master-mix for **Plate 1** in a 1.5 mL Eppendorf tube (adjust if not using *Phusion*):

	16S or 18S standard		18S with blocking primer	
	per 25 µL rxn	×100 rxns	per 25 µL rxn	×100 rxns
5× HF PCR Buffer	5 µL	500 µL	5 µL	500 µL
dNTPs (40 mM)	0.5 µL	50 µL	0.5 µL	50 µL
F primer (1 µM)	5 µL	} added	5 µL	} added
R primer (1 µM)	5 µL	} after	5 µL	} after
Blocking primer (10 µM)	-	-	4 µL	400 µL
Phusion (2 U/µL)	0.25 µL	25 µL	0.25 µL	25 µL
PCR-grade Water	7.25 µL	725 µL	3.25 µL	325 µL
Template	2 µL	added after	2 µL	added after

2.2 Dispense 78.5 µL of the master-mix into the 16 wells of 2 columns (or 105 µL in 12 wells of one row) of a 96-well plate (remaining wells to be used in subsequent PCR preps) – this plate now becomes the **Master-Mix Plate** and is used to transfer the master-mix into the PCR plate using an MCP.

2.3 Dispense 13 µL of master-mix into each well of the **PCR Plate 1 (2 µL)**, one column (or row) at a time with the MCP.

2.4 Remove the protective film ("uncover") from one column of the **Forward Set 1 Primer Plate**, align it horizontally on the bench to the left of **PCR Plate 1 (2 µL)** and dispense 5 µL into each well, one column at a time using the MCP. *Note: You can use the same set of 8 tips for all.*

2.5 Uncover one row of the **Reverse Set 1 Primer Plate**, align it vertically on the bench along the top of **PCR Plate 1 (2 µL)** and dispense 5 µL into each well, one row at a time using the MCP. *Note: You **must** now change tips after every row to avoid cross-contamination (since different F primers/indices are now in each row).*

2.6 Uncover the **DNA Plate 1**, align it along the top of **PCR Plate 1 (2 µL)** and dispense 2 µL into each well, one column at a time using the MCP. *Note: Remember to **change** tips after every column.*

2.7 Once complete, seal the plate with PCR film, place in a thermocycler and run the following program (~1.5 h; adjust if not using *Phusion*):

	w/o BP		with BP		
Initial denaturation	98°C	30 s	98°C	30 s	
Denaturation	98°C	10 s	98°C	10 s	30 cycles
Blocking primer annealing	-	-	70°C	30 s	
Annealing	55°C	30 s	55°C	30 s	
Extension	72°C	30 s	72°C	30 s	
Final Extension	72°C	4:30	72°C	4:30	
Hold	4°C	∞	4°C	∞	

2.8 While the first PCR is running, prepare the 1/10th dilution of **DNA Plate 1** – add 27 µL of PCR-grade water to each well of the remaining 3 µL of template in **DNA Plate 1**, for a total of 30 µL final volume per well, using a reservoir (~3 mL required) and MCP. *Note: Remember to **change** tips after every column.*

2.9 Once the first PCR is complete (or nearly so), repeat steps **2.1-2.7** to prepare **PCR Plate 1 (0.2 µL)** using the newly diluted templates. *Optional: Here, and for the subsequent plates below, once the two PCRs of the same plate are complete you can proceed to steps **3.1-3.4** and verify the PCR products before continuing with the next plate each time.*

2.10 Once the two PCRs for **Plate 1** are complete, repeat steps **2.1-2.9** to prepare **PCR Plates 2 (2 µL) & (0.2 µL)** from **DNA Plate 2** using **Forward Set 1 Primer Plate** and **Reverse Set 2 Primer Plate** (note change to F1+R2 here).

2.11 Once the two PCRs for **Plate 2** are complete, repeat steps **2.1-2.9** to prepare **PCR Plates 3 (2 µL) & (0.2 µL)** from **DNA Plate 3** using **Forward Set 2 Primer Plate** and **Reverse Set 1 Primer Plate** (note change to F2+R1 here).

2.12 Once the two PCRs for **Plate 3** are complete, repeat steps **2.1-2.9** to prepare **PCR Plates 4 (2 µL) & (0.2 µL)** from **DNA Plate 4** using **Forward Set 2 Primer Plate** and **Reverse Set 2 Primer Plate** (note change to F2+R2 here).

Step 3 – Gel Verification (2 h)

3.1 Plug in the *Mother E-Base*, unwrap a fresh *E-Gel 96* and insert it into the base.

3.2 The duplicate PCR reactions of **Plate 1** are aggregated then loaded onto the gel in the same action: using the MCP and working by rows (the gel cannot be loaded by columns as they are staggered), pipet 20 µL out of the **PCR Plate 1 (0.2 µL)** into the corresponding wells of **PCR Plate 1 (2 µL)** and mix by pipetting up and down, then take 20 µL of this aggregate and load into the appropriate wells of the gel. *Note: Remember to **change** tips after every row.* Discard the empty **PCR Plate 1 (0.2 µL)** when finished and relabel the **PCR Plate 1 (2 µL)** the **Aggregated PCR Plate 1**.

3.3 Once all rows are complete, load 20 µL of the *E-Gel Low Range Ladder* into some of the marker ("M") wells, then run the gel for the pre-set 12 min.

3.4 Visualize the gel and photograph on a UV/blue transilluminator with a SYBR filter. Example gels of 16S and 18S amplicons from this protocol are included at the end of this document for reference.

3.5 Repeat steps **3.1-3.4** for **PCR Plates 2 (2 µL) & (0.2 µL)**.

3.6 Repeat steps **3.1-3.4** for **PCR Plates 3 (2 µL) & (0.2 µL)**.

3.7 Repeat steps **3.1-3.4** for **PCR Plates 4 (2 µL) & (0.2 µL)**.

3.8 Any samples with failed PCRs (or spurious bands) are re-amplified by optimizing the PCR (further template dilution to 1:100 or using BSA/other additives) to produce correct bands in order to complete the amplicon plate. Unless this represents the majority of a plate (in which case continue with plates and E-gels), PCRs are done in standard tubes/strips and visualized using a traditional gel box. Once correct bands have been obtained, amalgamate those few tubes into the appropriate wells of the respective **Aggregated PCR Plates** before continuing.

Step 4 – PCR Clean-up + Normalization & Final Library Pool (2 h)

4.1 Use the remaining 20 µL of each well in the **Aggregated PCR Plate 1** to cleaned-up and normalize the amplicons using the high-throughput *Invitrogen SequalPrep 96-well Plate Kit*. Label this final plate **SequalPrep Plate 1**. *Note: We have found it more convenient to inverse steps 1 & 2 of the* SequalPrep *protocol (i.e.: buffer first with one set of tips, then amplicons with another set). Only one set of tips (per step) is need in this kit as carry-over is minimal, amplicons are barcoded (therefore can't "contaminate" each other's reads) and amplicons will shortly be pooled anyways.*

4.2 Once the *SequalPrep* protocol is complete, pool the 95 samples from **SequalPrep Plate 1** by using the MCP to transfer 5 µL of each column into one column of a new 96-well plate named the **Library Pool Plate** (remaining columns to be used in subsequent pooling). *Note: You can use the same set of 8 tips for all.* Once complete, pipette 50 µL of each of the 8 wells into one 1.5 mL Eppendorf tube and label **Plate 1 Library Pool**.

4.3 Repeat steps **4.1 & 4.2** for **Aggregated PCR Plate 2**.

4.4 Repeat steps **4.1 & 4.2** for **Aggregated PCR Plate 3**.

4.5 Repeat steps **4.1 & 4.2** for **Aggregated PCR Plate 4**.

4.6 Once all four pools are complete, pipette 100 µL of each of the four tubes into one 1.5 mL Eppendorf tube and label **Final Library Pool** (add the run name to the tube or some other identifier to keep your various pools separate).

4.7 Quantify the **Final Library Pool** using the *Invitrogen Qubit dsDNA HS* assay (or similar fluorescence-based alternative; 5 µL of pool to be assayed) and calculate the molar concentration using the following formula, knowing that 1 ng/µL of a 500 bp amplicon = 3.29 nM:

$$\left(\frac{500\ bp}{size\ in\ bp\ of\ amplicon}\right) \times (concentration\ in\ ng/\mu L) \times (3.29)$$

For the 16S amplicon generated using our V6-V8 primers (= 574 bp, including target region + adaptors + indices) at a concentration of 3.0 ng/µL, for example:

$$\left(\frac{500\ bp}{574\ bp}\right) \times (3.0) \times (3.29) = 8.60\ nM$$

Note: We have found that the anticipated 1-2 ng/µL output from the *SequalPrep* plate is near impossible to achieve; we typically see concentrations in the range of 0.3-0.9 ng/µL.

Step 5 – *Illumina MiSeq* Sequencing (~3 days [only ~2 h hands-on at the start and end])

5.0 This section is based upon the following *Illumina* documents, with some small procedural changes (including using the *NextSeq* variant for sample denaturation), and the inclusion of instructions to be able to load >96 samples (i.e.: 384 combinations of indices) which are not written out by *Illumina* – familiarize yourself with these documents / have them on-hand:

- *MiSeq Reagent Kit v3 – Reagent Preparation Guide*
- *Preparing Libraries for Sequencing on the MiSeq*
- *Denaturing and Diluting Libraries for the NextSeq 500*
- *MiSeq System User Guide*

5.1 Begin thawing the *v3 Reagent Cartridge* and tube of *HT1* as instructed. Put at 4°C when complete. *Optional: Take out the day before and thaw the reagents overnight at 4°C.*

5.2 While waiting, prepare the *Sample Plate* and *Sample Sheet* files that will be used to run the *MiSeq* by opening the *Illumina Experiment Manager (iEM)* software.

5.3 Create the *Sample Plates* first – in order to run all 384 combinations of indices, 4 separate *Sample Plates* (one per plate from our protocol above) will be required.

For the samples that were in **DNA Plate 1**:

- Choose *Nextera XT v2 (Set A)* in the *iEM* wizard.
- Give the plate a unique name (we usually use our run number and append an "A" to the end; ex.: *15A*).
- Copy-and-paste the 96 sample names from your Excel sheet (after having brought the file over to the *MiSeq* via USB, Dropbox or email) into the *Plate* tab, then press *Apply Default Index Layout*. You will sometimes not see the index names show up on the top row and left-hand column of this tab, but if you switch to the *Plate Graphic* or *Table* views, they will be there correctly.
- Click on *Finish* and save the **A.nexxt28.plt* file in the directory of your choice.

5.4 For the samples that were in **DNA Plate 2**, repeat step **5.3** except change to *Nextera XT v2 (Set B)* and append a "B" to the filename.

5.5 For the samples that were in **DNA Plate 3**, repeat step **5.3** except change to *Nextera XT v2 (Set C)* and append a "C" to the filename.

5.6 For the samples that were in **DNA Plate 4**, repeat step **5.3** except change to *Nextera XT v2 (Set D)* and append a "D" to the filename.

5.7 Now create the *Sample Sheet* by bringing in the 4 *Sample Plates* that belong to it:

- Select *MiSeq* in the *iEM* wizard, then *Other* → *FASTQ Only*.
- Input your Reagent Cartridge barcode, select *Nextera XT v2* for the Sample Prep Kit, input your Experiment Name (we usually use our complete run name; ex.: *IMR-Run15*) and change the cycles to 301 for both reads.
- On the next screen, uncheck the *Maximize* box, choose *Select Plate* at left and navigate to and select your **A.nexxt28.plt* file (*Plate A*).
- Once the samples are displayed, choose *Select All* + *Add Selected Samples*.
- Repeat the above two steps for the remaining plate files (*Plates B/C/D*).
- The Sample Sheet Status should show as Valid and, if so, click *Finish* to save the file, appending the run name to the end of the filename (for the above ex.: *MSxxxxxxx-600V3-Run15.csv*). We find it helpful having the run name/# when returning to the files, otherwise they are only labeled with the less-informative cartridge barcode by default. If your Sample Sheet Status is showing as Invalid, then it is often due to identical sample names being used in error. Once corrected, the status will update.
- It is a good idea to then simply verify that the CSV file is all correct by opening the file in *WordPad* and checking that the header information is correct (date, run name, FASTQ generation, etc.) and that you see the four sets of samples below in the [Data] section (*Plate A* samples, followed by *Plate B*, etc.).

5.8 Prepare 0.2 N NaOH as instructed, except make 10-fold less (20 µL of 1 N NaOH + 80 µL of water).

5.9 Use the nanomolar concentration from step **4.7** to determine whether the final amplicon library must be diluted or concentrated prior to continuing. A fixed

concentration of 4 nM is the standard requirement for the *MiSeq*, however, using the *NextSeq* loading protocol, a library between 0.4-4 nM can be accommodated by simply using a larger volume of a more dilute library. Use the following guidelines:

**[Library]**	**Vol. to denature**	**0.2 N NaOH**	**Tris-HCl**	**HT1**
>4 nM	*dilute to 4 nM using PCR-grade water or buffer*			
4 nM	5 µL	5 µL	5 µL	985 µL
2 nM	10 µL	10 µL	10 µL	970 µL
1 nM	20 µL	20 µL	20 µL	940 µL
0.5 nM	40 µL	40 µL	40 µL	880 µL
0.4 nM	50 µL	50 µL	50 µL	850 µL
<0.4 nM	*concentrate using stand. method/kit & repeat step* ***4.7***			

5.10 Denature the library, using the indicated volume of 0.2 N NaOH in the table above, for 5 min. at room temperature.

5.11 Neutralize the reaction by adding the equivalent volume of Tris-HCl as indicated.

5.12 Dilute out the library to 20 pM using the indicated amount of chilled *HT1* and place on ice.

5.13 Combine 570 µL of the library with 30 µL (= 5%) of the already diluted and denatured *PhiX Control Library*. We always use 5% with amplicon libraries, regardless of anticipated diversity.

5.14 Proceed with loading the 600 µL sample in the *v3 Reagent Cartridge* and continue the *MiSeq* run start procedure, as instructed – the only slight change is that the default filename (just the cartridge barcode) of the *Sample Sheet* will not be found since we appended the run name to the end of the CSV file (for the above ex.: *MSxxxxxxx-600V3-Run15.csv*); browse to and select the correct file.

Example *E-gel 96* Image of 16S Amplicons from this Protocol (all positive; *E-gel Low Range Ladders* absent from marker lanes)

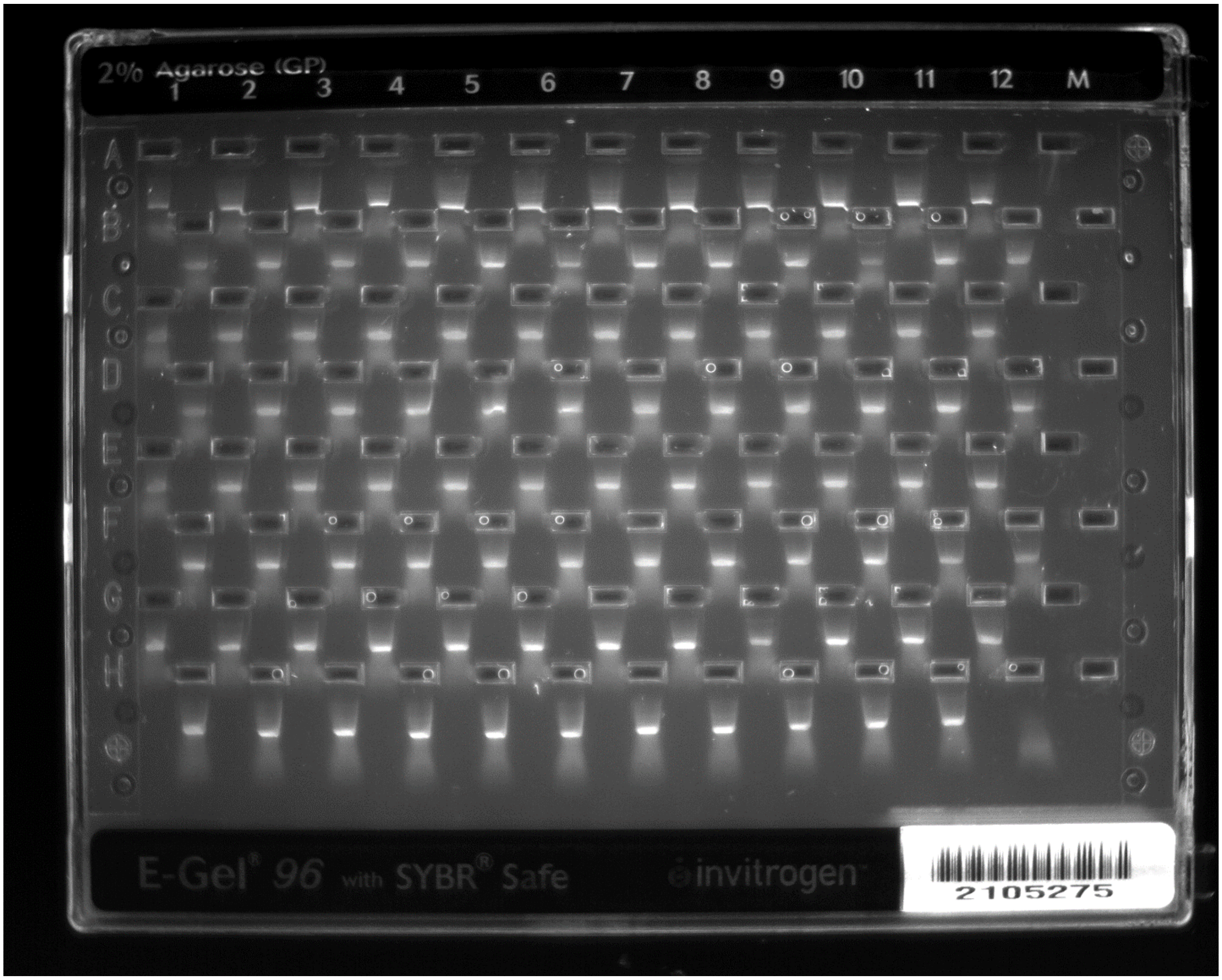

EXAMPLE *E-GEL 96* IMAGE OF 16S AMPLICONS FROM THIS PROTOCOL (SOME WEAK/NEGATIVE; *E-GEL LOW RANGE LADDERS* ABSENT FROM MARKER LANES)

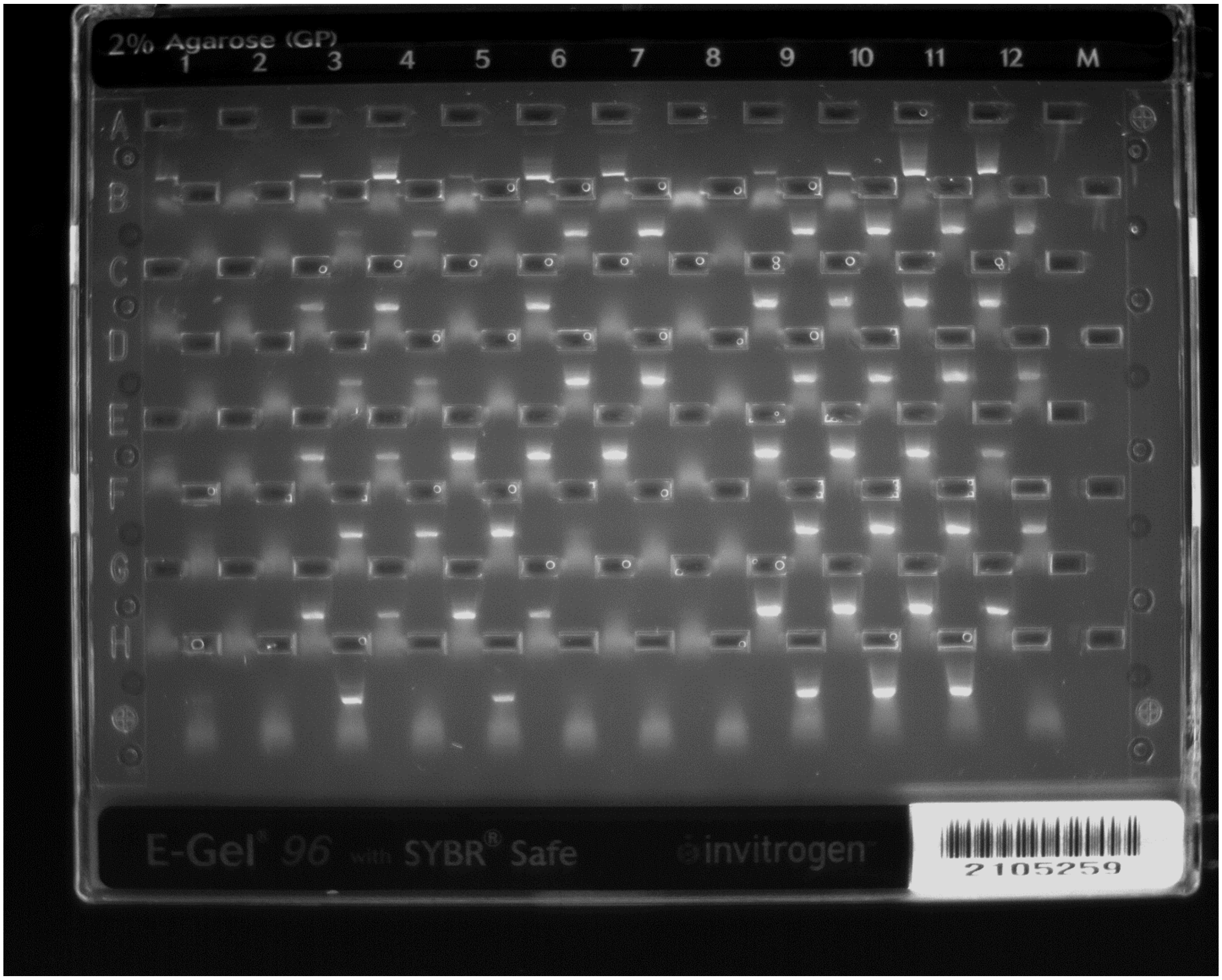

EXAMPLE *E-GEL 96* IMAGE OF 18S AMPLICONS FROM THIS PROTOCOL (ALL POSITIVE; *E-GEL LOW RANGE LADDERS* ABSENT FROM MARKER LANES) – NOTE THE EXTRA FAINT BANDS ABOVE THE MAIN AMPLICONS REPRESENTING THE TEMPLATES WITH INSERTIONS IN THEIR V4 REGIONS

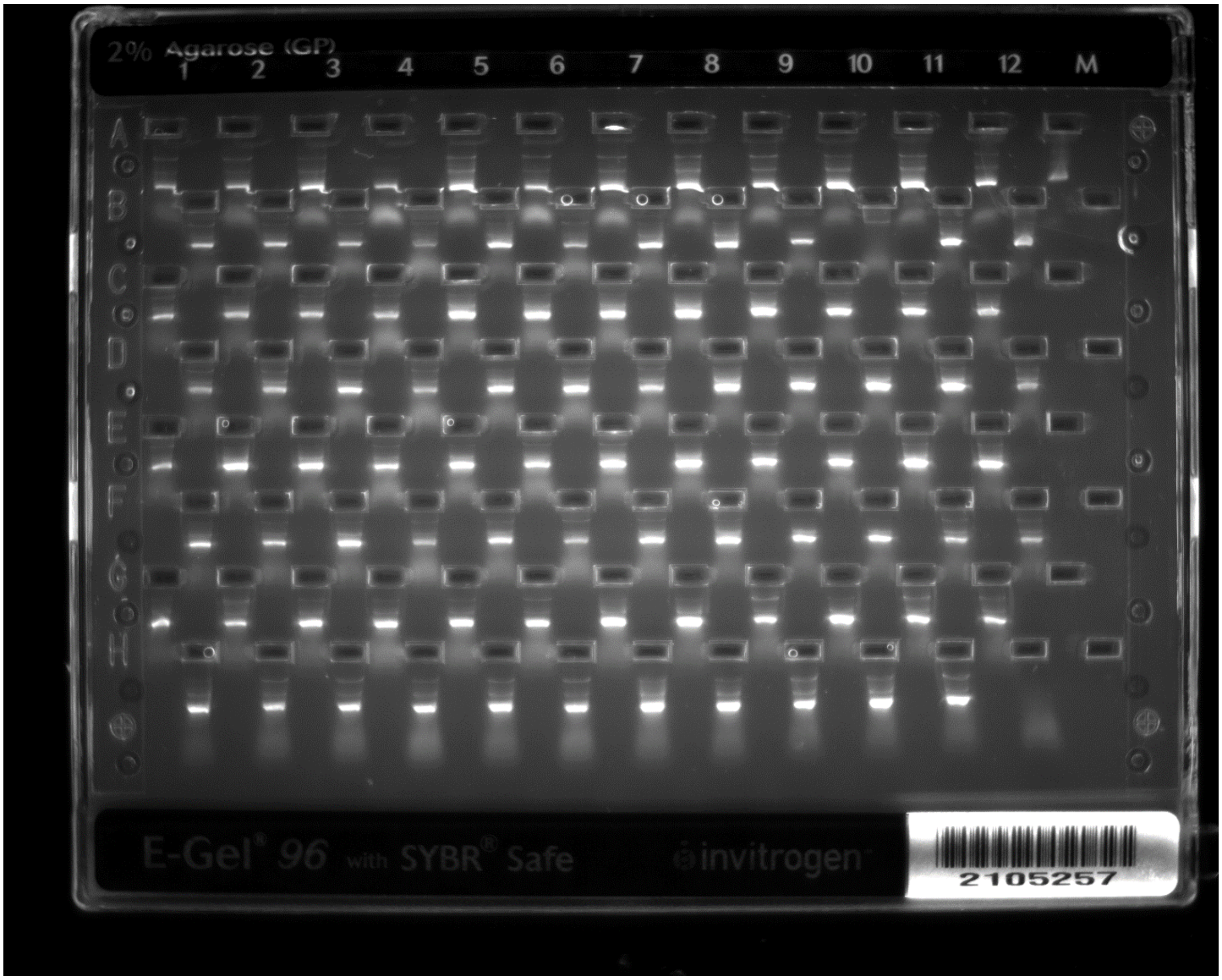

Supplement: TEXT S1 [file sys001172076s1.pdf]
